# DNA-Based Nanobiosensor for the Colorimetric Detection of Dengue Virus Serotype 2 Synthetic Target Oligonucleotide

**DOI:** 10.3390/bios15020071

**Published:** 2025-01-24

**Authors:** Michael Sandino C. Flores, Evangelyn C. Alocilja, Divina M. Amalin, Mae Joanne B. Aguila, Marynold V. Purificacion, Florinia E. Merca, Ma. Carmina C. Manuel, Mark Pierre S. Dimamay, Ma. Anita M. Bautista, Lilia M. Fernando

**Affiliations:** 1 Institute of Chemistry, College of Arts and Sciences, University of the Philippines Los Baños, College, Los Baños 4031, Philippines; mbaguila@up.edu.ph (M.J.B.A.); mvpurificacion@up.edu.ph (M.V.P.); femerca@up.edu.ph (F.E.M.); 2 Nano-Biosensors Lab, Biosystems and Agricultural Engineering, Michigan State University, East Lansing, MI 48824, USA; alocilja@msu.edu; 3 Global Alliance for Rapid Diagnostics, Michigan State University, East Lansing, MI 48824, USA; 4 Department of Biology, College of Science, De La Salle University Manila, Taft Avenue, Malate, Manila 1004, Philippines; divina.amalin@dlsu.edu.ph; 5 Institute of Biological Control, De La Salle University-Laguna Campus, Biñan City 4024, Philippines; 6 Institute of Plant Breeding, College of Agriculture and Food Science, University of the Philippines Los Baños, College, Los Baños 4031, Philippines; 7 Genetics and Molecular Biology Division, Institute of Biological Sciences, College of Arts and Sciences, University of the Philippines Los Baños, College, Los Baños 4031, Philippines; mcmanuel1@up.edu.ph; 8 Research and Biotechnology Division, St. Luke’s Medical Center, Quezon City 1112, Philippines; mpsdimamay@stlukes.com.ph; 9 Functional Genomics Laboratory, National Institute of Molecular Biology and Biotechnology, University of the Philippines Diliman, Quezon City 1101, Philippines; mmbautista20@up.edu.ph; 10 Institute of Crop Science, College of Agriculture and Food Science, University of the Philippines Los Baños, College, Los Baños 4031, Philippines

**Keywords:** colorimetric detection, dengue virus serotype 2, dextrin-capped gold nanoparticles, nanobiosensor

## Abstract

Annually, the Philippines is burdened by a high number of infections and deaths due to Dengue. This disease is caused by the Dengue virus (DENV) and is transmitted from one human host to another by the female *Aedes aegypti* mosquito. Being a developing country, most of the high-risk areas in the Philippines are resource-limited and cannot afford equipment for detection and monitoring. Moreover, traditional clinical diagnoses of DENV infection are costly and time-consuming and require expertise. Hence, it is important to establish effective vector control and surveillance measures. In this study, we developed a DNA-based nanobiosensor for the colorimetric detection of Dengue virus serotype 2 (DENV-2) synthetic target DNA (stDNA S2) using gold nanoparticles (AuNPs). We successfully functionalized dextrin-capped gold nanoparticles with the designed DENV-2 oligonucleotide probes. The detection of the complementary stDNA S2, indicated by the pink-colored solution, was successfully performed within 15 min using 0.40 M NaCl solution. We were able to detect up to 36.14 ng/μL of stDNA S2 with some cross-reactivity observed with one non-complementary target. We believe that our study offers a basis for developing nanobiosensors for other DENV serotypes.

## 1. Introduction

Dengue is a mosquito-borne disease that poses a significant global health challenge, particularly in tropical and subtropical regions [1,2,3]. In 2023, a record high of over 6.5 million Dengue cases was reported, resulting in approximately 7300 deaths across more than 80 countries [4]. The surge in infections has been exacerbated by factors such as climate change, the coronavirus disease 2019 (COVID-19) pandemic, and various political and financial instabilities in affected regions [4]. It is estimated that around 390 million people are infected with the virus each year, leading to about 20,000 deaths due to Dengue hemorrhagic fever (DHF) annually [5,6].

In the Philippines, which is situated in a tropical region, the burden of Dengue infections and related deaths is particularly high annually. The Epidemiology Bureau of the Department of Health (DOH) considers Dengue as one of the ten leading causes of morbidity in the country, with a rate of 48.38 per 100,000 population [7]. In 2018, the number of Dengue cases in the Philippines peaked at 51,361, ranking it as the ninth leading cause of morbidity [8]. The following year saw the declaration of an outbreak, marking the worst Dengue surge recorded since 2012, with a total of 437,563 cases and 1689 deaths reported by the DOH [9]. The rise in cases, seen not only in rural areas but also in densely populated urban centers, can be attributed to rapid population growth and urbanization, ecological factors such as temperature, rainfall, and altitude, virus and vector evolution, ineffective vector control measures, and increased air transport globalization [2,10,11].

The Dengue virus (DENV), a member of the *Flavivirus* genus, is the causative agent of Dengue. DENV has a positive-sense single-stranded RNA as its genetic material [(+)ssRNA] [12,13]. There are four genetically related but distinct (4) antigenic groups or serotypes of this virus, namely Dengue virus serotype 1 (DENV-1), Dengue virus serotype 2 (DENV-2), Dengue virus serotype 3 (DENV-3), and Dengue virus serotype 4 (DENV-4) [14]. DENV-3 was previously known as the most common Dengue serotype in the Philippines [11,15]; however, recent studies have indicated that both DENV-1 and DENV-2 are also circulating widely, depending on the year, especially between 2015 and 2017 [16]. Infection with one serotype does not guarantee immunity against the others, and secondary infections are often more severe for individuals who have been previously infected.

The primary vector for Dengue is the day-biting female *Aedes aegypti* mosquito, which is especially rampant in tropical regions like the Philippines [11]. DENV present in the salivary glands of the mosquito is injected into the host when they feed through sucking blood [12]. Infected individuals may experience Dengue fever (DF), DHF, or Dengue shock syndrome (DSS) [3]. Current diagnostic methods for confirming Dengue include viral isolation, serological testing, and molecular diagnostics. Unfortunately, these methods are often complex, time-consuming, labor-intensive, and costly and require specialized expertise [12,17]. The gold standard for sensitive molecular diagnosis of Dengue is the reverse transcriptase–polymerase chain reaction (RT-PCR), but this method is expensive and not widely available in endemic areas [3].

According to the World Health Organization (WHO), effective vector control and surveillance are crucial in combating the transmission of Dengue [18]. Hence, vector surveillance measures aimed at detecting DENV in mosquitoes can facilitate early detection and prevention of future outbreaks in communities [15,19]. However, many high-risk areas in the Philippines face resource limitations, making it challenging to access necessary detection and monitoring equipment.

In recent years, rapid diagnostic methods for DENV using biosensors have been developed, which could help address these challenges. Table 1 summarizes various biosensors that have been developed to detect DENV.

Gold-nanoparticle-based colorimetric biosensors have gained significant attention in recent years due to their ability to rapidly detect various pathogens with low detection limits [15]. Additionally, this method is straightforward and more affordable compared to conventional pathogen detection methods and offers portability for field applications [3]. The unique physicochemical and optical properties of AuNPs, which are different from their bulk counterpart, arise from localized surface plasmon resonance (LSPR). This phenomenon occurs when the 6s electrons (plasmons) in the conduction band of AuNPs interact with the electromagnetic field of incident light. When the frequency of the collective oscillations of these conduction band electrons matches the frequency of the incident light, resonance coupling takes place, leading to the absorption of the radiation and giving rise to absorption bands [24,25].

Due to LSPR, AuNPs exhibit strong absorption in the ultraviolet-visible (UV-Vis) region [25]. The wavelength of maximum absorption (λ_max_) of AuNPs is influenced by their size, shape, and interparticle distance [25]. Specifically, for colloidal solutions of AuNPs measuring 13 to 20 nm, the λ_max_ (also known as the LSPR band or peak) is approximately 520 nm and has a wine-red color [25,26]. However, when the interparticle distance of the AuNPs decreases (indicating aggregation) or when the size of the particles increases, a significant redshift occurs, along with the broadening of the LSPR band [25]. The plasmon peak at 520 nm gradually diminishes, resulting in a new plasmon peak emerging between approximately 600 and 700 nm. Consequently, the color of the colloidal AuNP solution transitions from red to purple or blue, depending on the degree of aggregation [24,25,26]. In extreme cases of aggregation, the AuNP colloidal solution may become colorless, as the plasmon peak shifts into the infrared (IR) region [24].

Leveraging the unique physicochemical and optical properties of AuNPs, colorimetric biosensors for DENV could be developed for the rapid, cost-effective, sensitive, and field-applicable detection of DENV in *Aedes aegypti* mosquitos. This nanobiosensor could aid in the effective monitoring and early detection of future Dengue outbreaks, particularly in high-risk, resource-limited communities in the Philippines. Herein, we developed a DNA-based nanobiosensor for the colorimetric detection of Dengue virus serotype 2 (DENV-2) synthetic target DNA (stDNA S2) using gold nanoparticles. To achieve this, we designed oligonucleotide probes for DENV-2 using a bioinformatics approach. Following this, the designed DENV-2 probes were immobilized on the prepared dextrin-capped gold nanoparticles. Then, the reaction conditions were optimized for the colorimetric detection of the stDNA S2. Finally, we evaluated the nanobiosensor’s sensitivity and specificity.

## 2. Materials and Methods

### 2.1. Reagents and Equipment

Dextrin from potato starch (C_6_H_12_O_6_), tris(2-carboxyethyl)phosphine hydrochloride (TCEP) (C_9_H_15_O_6_P · HCl), and gold(III) chloride trihydrate (HAuCl_4_) were purchased from Sigma-Aldrich (St. Louis, MO, USA). On the other hand, sodium carbonate (Na_2_CO_3_) and sodium chloride (NaCl) were sourced from Loba Chemie (Mumbai, India). Phosphate-buffered saline (PBS) and Tris-Ethylenediamine tetraacetic acid (TE) (C_10_H_16_N_2_O_8_) buffer were prepared using the reagents obtained from Sigma-Aldrich (St. Louis, MO, USA). Thiolated oligonucleotide (DNA) probes were purchased from Integrated DNA Technologies (IDT) (Singapore). Dithiothreitol (DTT) (C_4_H_10_O_2_S_2_) and ethyl acetate (CH_3_COOCH_3_) were purchased from RCI Labscan (Bangkok, Thailand), while sodium dodecyl sulfate (SDS) (NaC_12_H_25_SO_4_) was sourced from J.T. Baker (Allentown, PA, USA). The hybridization of the probe with the synthetic oligonucleotide target was facilitated by placing the reaction solutions in a ^3^PrimeG02 thermocycler from Techne (Ramsey, MN, USA). In addition, the UV-Vis spectrophotometric readings were conducted using a Multiskan GO spectrophotometer from Thermo-Scientific (Waltham, MA, USA). The measurement of the nanoparticle size was performed via dynamic light scattering (DLS) on an SZ-100 Nanopartica from Horiba (Singapore). Finally, scanning electron microscopy with energy-dispersive X-ray (SEM-EDX) was carried out using a Prisma™ E SEM from Thermo Fisher Scientific (Waltham, MA, USA).

### 2.2. Oligonucleotide Probe Designs

DENV-2 viral DNA sequences were retrieved from the National Center for Biotechnology Information (NCBI) Virus database using the following parameters: (a) serotype: DENV-2; (b) disease: Dengue; (c) host: *Aedes*: (d) region/country: Asia; (e) genome region: any; (f) isolation source: any; and (g) genome type: complete and incomplete (Table S1). Specifically, sequences for the envelope (*E*) gene of the virus were chosen as the target for the design of the probes. The *E* gene is the most common target for detection since it is highly conserved and is evolutionarily constrained since it is vital for the binding of the virus into the host cell—making it an ideal target for detection [27].

Multiple sequence alignment (MSA) of the retrieved seventy (70) *Aedes aegypti* DENV-2 DNA sequences was performed using MEGA10.0.5: Molecular Evolutionary Genetics Analysis software with the ClustalW algorithm [28]. Using Geneious Prime version 2020.0.5, two (2) conserved sequences having at least a 65% identity threshold, being more than 20 bases in length, and having at least 50% guanine + cytosine content were selected from the MSA [29]. Other physical properties of these selected conserved sequences were also determined using the OligoAnalyzer Tool feature of Integrated DNA Technologies (IDT): (1) melting temperature (T_m_ in °C); (2) molecular weight (in g/mole); (3) extinction coefficient (L/mol-cm); and (4) optical density (OD_260_; either in nmole or μg) [30]. Potential hairpins, self-dimers, and hetero-dimers of these sequences were also predicted using the hairpin, self-dimer, and hetero-dimer tools of IDT, respectively. The specificity of these conserved sequences as DENV oligonucleotide probes was evaluated using NCBI’s Basic Local Alignment Search Tool (BLAST) [31].

Between the two (2) conserved sequences, one was selected as a DENV-2 oligonucleotide probe (DENV-2 probe) with the following sequence: /5ThioMC6-D/AAAAAAAAAAAGCTGTAGCTTGTCCATTCTCAGCCTGCACTTG. Additional modifications, specifically 5′-end thiol modification and poly-A10 spacer, were added to the design of the probe. The 5′-end thiol modification (i.e., /5ThioMC6-D/) was essential for linking the probe to gold nanoparticles through a gold–sulfur bond. On the other hand, the poly-A_10_ spacer included in between the recognition sequence and the thiol modification was added to reduce the unwanted steric crowding on the surface of the gold nanoparticles, especially during the immobilization of the probes on the nanoparticle surface and during the hybridization of the probe with the target DNA sequence [32]. The complementary sequence of the DENV-2 probe was also designed as the synthetic target DNA (stDNA S2): GTCGTCAGGAAACTTGCTCTTCACTGGACATCTCAAGTGCAGGCTGAGAATGGACAAGCTACAGCTCAAAGGAATGTCATACTCTATGTGCA-CAGGAAAG. Both the DENV-2 oligonucleotide probe and stDNA S2 were synthesized at a commercial facility (Integrated DNA Technologies, Singapore).

### 2.3. One-Pot Alkaline Synthesis and Characterization of the d-AuNPs

Dextrin-capped gold nanoparticles (d-AuNPs) were prepared using the methods described by Anderson and colleagues [33]. Before the preparation of the nanoparticles, all glassware was thoroughly washed with aqua regia (3:1 concentrated HCl/concentrated HNO_3_) followed by rinsing with distilled water and distilled water (dH_2_O) type 1. In brief, 20 mL of sterile distilled water type 1 (sdH_2_O type 1) was transferred into a 50 mL sterile conical tube. Next, 20 mL of sterile 25 g/L dextrin was added into the tube (C_f_ = 10 g/L). This was followed by the addition of 5 mL of 8 g/L HAuCl_4_ solution. The pH of the solution was adjusted to pH 9.3 using sterile 10% (*w*/*v*) Na_2_CO_3_ solution followed by dilution to the mark (V_f_ = 50 mL) using sterile pH 9.0 water. The solution was transferred into a sterile 125 mL Erlenmeyer flask and was completely covered with aluminum foil. It was incubated for 6 h at 50 °C with continuous shaking at 100 revolutions per minute (rpm).

The absorbance spectrum (400–700 nm) of the d-AuNP solution was generated and the absorbance at λ_max_ = 520 nm and λ = 620 nm was determined using a UV-Vis spectrophotometer. Dynamic light scattering (DLS) using a Zetasizer (particle size analyzer) was performed to assess the average particle size, polydispersity index (PDI), and zeta (ζ) potential of the same d-AuNP solutions. Three replicate measurements were conducted for each dilution, and the average values were calculated. Lastly, SEM-EDX was employed to obtain images of the d-AuNPs and to confirm their morphology and spherical structure. Approximately 20 µL of the diluted samples was placed onto a lamella for this analysis.

### 2.4. Conjugation of the Oligonucleotide Probes with the d-AuNPs

The functionalization of the d-AuNPs with the DENV-2 probe was carried out using the salt-aging method described previously [32,34,35]. To activate (i.e., reduce) the DENV-2 probe, 40 µL of 100 µM DENV-2 probe was mixed with 40 µL of 10 mM TCEP solution (1:100 DENV-2 probe/TCEP) in a sterile 1.5 mL tube. The tube was wrapped in aluminum foil, and the reaction was allowed to stand at room temperature for 2 h.

Next, 20 µL of the previously reduced thiolated DENV-2 probes at 0.05, 0.10, 0.50, 1.00, and 2.00 nmol was mixed with 1.0 mL of d-AuNPs in 1.5 mL tubes. The tubes were again covered with aluminum foil and incubated overnight (~12 h) at room temperature. Afterward, a phosphate adjustment buffer (PAB) containing 100 mM phosphate buffer and surfactant solution [10% (*w*/*v*) SDS] was added into the solutions to achieve a final concentration of 9 mM and 0.1% (*w*/*v*), respectively. The mixtures were allowed to incubate for an additional 30 min at room temperature.

The concentration of NaCl was then gradually increased to 0.3 M through six (6) successive additions of the salting buffer [10 mM phosphate buffer + 2 M NaCl (pH 7.0)]. The reaction mixture was sonicated for approximately 10 s with every addition of the salting buffer and was allowed to stand at room temperature for an hour. After the final addition, the solutions were equilibrated overnight at room temperature. The unreacted probes and TCEP were removed by centrifugation at 10,000 rpm for 15 min and 15 °C. The supernatant was carefully discarded while ensuring that the d-AuNP/DENV-2 probes were washed with washing buffer [0.01% (*w*/*v*) SDS] two (2) times. It was important to disperse the pellets during washing to prevent irreversible aggregation. Finally, the d-AuNP/DENV-2 probes were resuspended in 0.01% (*w*/*v*) SDS solution.

### 2.5. Optimization of the Detection

Colorimetric detection was performed by measuring the response (A_520_/A_620_) of the 1.00 nmol d-AuNP/DENV-2 probes in NaCl solutions with final concentrations of 0.0, 0.10, 0.20, 0.30, 0.40, 0.50, 0.60, 0.70, 0.80, 0.90, 1.00, 1.20, 1.30, 1.40, and 1.50 M. The solutions were composed of 10 µL of d-AuNP/DENV-2 probes, 10 µL of sterile phosphate-buffered saline (sPBS), 30 µL of sdH_2_O type 1, and 50 µL of NaCl solution. The absorbance at 520 nm and 620 nm of these solutions was read in triplicate using a UV-Vis spectrophotometer after a 15-min incubation period.

### 2.6. Detection of the Synthetic Oligonucleotide Targets Using the Optimized Conditions

The colorimetric detection of the stDNA S2 using a 1.0 nmol d-AuNP/DENV-2 probe was conducted using the previously optimized conditions. Blank 1 (without NaCl), blank 2 (with NaCl), and samples containing complementary and non-complementary targets were prepared as outlined in Table 2.

The necessary components were mixed in 0.2 mL tubes and placed in a thermocycler, where they underwent one cycle consisting of denaturation at 95 °C (5 min), annealing at 55 °C (10 min), and cooling at 25 °C (5 min). After cooling to room temperature (28 to 30 °C), 50 µL of 0.80 M NaCl solution was added to both negative and positive samples, while sdH_2_O type 1 was added to the blank. The solutions were mixed thoroughly by pipetting and incubated for 15 min at room temperature before measuring absorbance at 520 nm and 620 nm using a UV-Vis spectrophotometer. The response (A_520_/A_620_) of these solutions was determined.

### 2.7. Sensitivity and Specificity Assays

To evaluate the sensitivity of the optimized colorimetric detection method, stDNA S2 solutions of varying concentrations (100, 10, 1.0, 0.1, 0.01, and 0.001 µM) were prepared through serial dilution in 1X sPBS. On the other hand, the specificity was determined by testing the d-AuNP/DENV-2 probe against non-complementary synthetic target DNAs, specifically (a) stDNA PP (*Phytophthora palmivora*) and (b) stDNA LT (*Lasiodiplodia theobromae*). The procedure for detecting stDNA S2 in positive samples was similarly applied for the sensitivity and specificity assays (as detailed in Table 2). The response (A_520_/A_620_) of these solutions was determined in triplicate (3).

### 2.8. Statistical Analyses

The mean or average absorbance at 520 nm and 620 nm of the three (3) replicates was determined using Microsoft^®^ Excel^®^ (MS Excel) for Microsoft 365. One-way analysis of variance (ANOVA) and a Tukey Honestly Significant Difference (HSD) Test (post hoc test) at α = 0.05 were performed using IBM^®^ SPSS^®^ Statistics version 26.0.0.0. Finally, graphs with error bars (i.e., standard errors) and significant difference labels (i.e., different letters indicate significant differences) were constructed using Microsoft^®^ Excel^®^ (MS Excel) for Microsoft 365.

## 3. Results

### 3.1. Synthesis and Characterization of the d-AuNPs

The HAuCl_4_, sdH_2_O type 1, and dextrin mixture appeared as a clear yellow solution with an initial pH of 2.8. Upon adjustment from pH 2.8 to pH 9.0 using a 10% (*w*/*v*) Na_2_CO_3_ solution, the color of the solution changed to gray. After six (6) hours of incubation at 50 °C with shaking (100 rpm), the color of the solution changed to wine red. These color changes were consistent with those described by Anderson and colleagues, indicating the successful preparation of the d-AuNPs [33].

The absorbance spectra of the as-synthesized d-AuNP solutions indicated that the wavelength of maximum absorption was approximately 520 nm, and this is consistent with previous research [15,33,36,37]. For the DLS spectroscopy, the average size obtained for the 1:9, 1:49, and 1:99 d-AuNP dilutions were 19.4 ± 0.6 nm, 18.7 ± 2.4 nm, and 21.0 ± 2.4, respectively. The average sizes of the d-AuNPs across the three dilutions were not significantly different from one another. Furthermore, these values were relatively close to the 10.6 ± 1.6 nm reported by Anderson and co-researchers when they implemented this method [33].

PDI is a measure of the size heterogeneity, whether uniform or non-uniform, of the nanoparticles in a solution [38]. In this case, the following PDI values are reported for the 1:9, 1:49, and 1:99 d-AuNP dilutions: 0.242 ± 0.034, 0.346 ± 0.112, and 0.228 ± 0.083, respectively. Again, there were no significant differences in the PDI among the three dilutions. The low PDI values suggest that the d-AuNPs are relatively uniform in size.

The zeta (ζ) potential indicates the colloidal stability of a solution containing charged particles and is related to the net surface charge of nanoparticles. A sufficiently high zeta potential of the same sign—in this case, a negative sign—was indicative of greater stability and a reduced tendency for the nanoparticles to aggregate [39]. The obtained zeta (ζ) potentials were −14.4 ± 69.4, −21.0 ± 61.7, and −45.2 ± 62.4 mV for the 1:9, 1:49, and 1:99 d-AuNP dilutions, respectively. These negative values indicate that the d-AuNP colloidal system is stable. Statistical analysis revealed no significant differences between the zeta (ζ) potentials.

**Figure 1:**
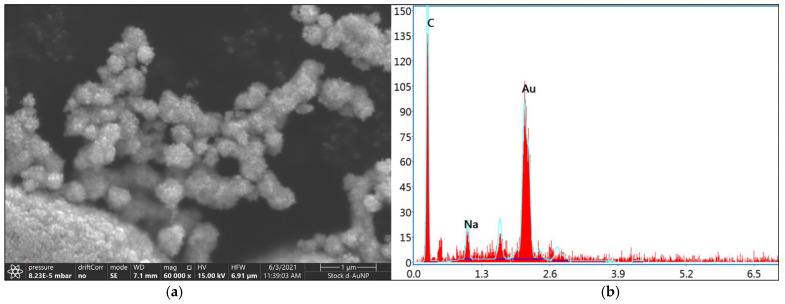
The (**a**) SEM image of the prepared d-AuNPs at 60,000× magnification and (**b**) the EDX spectra of a selected area of the sample. The element gold had a weight percentage of 52.99%.

SEM images captured at 60,000× magnification confirmed the spherical structures of the d-AuNPs (Figure 1a). Based on the energy-dispersive X-ray (EDX) spectra, the selected area showed four counts of the following elements: carbon (C), sodium (Na), oxygen (O), and gold (Au) (Figure 1b).

Among these, gold was the predominant element, comprising 52.99% by weight, followed by carbon at 43.18% and sodium at 3.83%. The EDX spectroscopy results confirmed the incorporation of gold into the d-AuNP samples.

### 3.2. Immobilization of the Oligonucleotide Probes on the d-AuNPs

**Figure 2:**
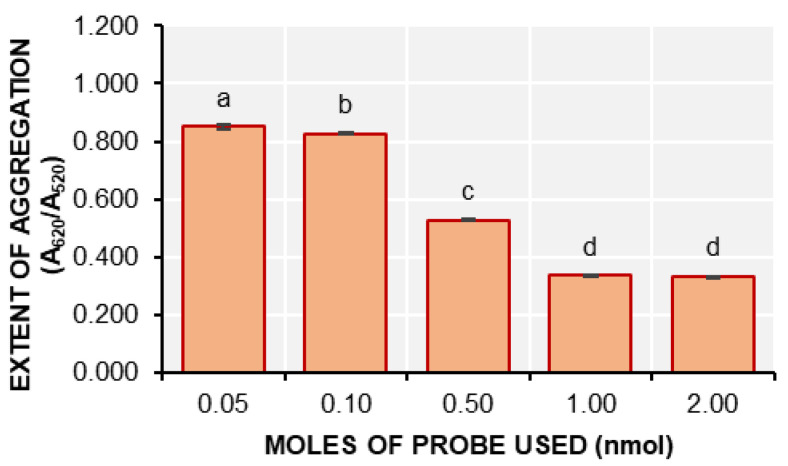
The extent of aggregation (A_620_/A_520_) of the d-AuNP/DENV-2 probes after salt-aging (mean ± SD; *n* = 3). Samples with different letters on top of the bars are significantly different based on ANOVA and Tukey HSD test at α = 0.05.

At lower amounts of DENV-2 probes, specifically 0.05 nmol, 0.1 nmol, and 0.5 nmol, the d-AuNP/DENV-2 probes aggregated following the salt-aging method. This aggregation was evident in the formation of precipitates and the violet color observed in the solution. Further verification of this phenomenon was conducted using UV-Vis spectrophotometry (Figure 2).

The high extent of aggregation (A_620_/A_520_) of the immobilization solutions which contained 0.05 and 0.10 nmol of DENV-2 probes was also significantly higher (*p* < 0.05) than those which contained 0.50, 1.00, and 2.00 nmol of the probes. These findings confirm that aggregation occurs in the solutions with 0.05 and 0.10 nmol of DENV-2 probes. Additionally, the precipitates became more pronounced after purifying the conjugation solutions that contained 0.05, 0.10, and 0.50 nmol of DENV-2 probes.

In contrast, no visible aggregates were observed in the solutions containing 1.00 and 2.00 nmol of DENV-2 probes. This suggests that 1.00 or 2.00 nmol of DENV-2 probes is sufficient to maintain the stability of the conjugates and prevent aggregation. Therefore, it can be concluded that the lowest concentration of DENV-2 probes that can be added to 1.0 mL of d-AuNPs without causing aggregation is 1.0 nmol.

### 3.3. Optimization of the Colorimetric Detection

**Figure 3:**
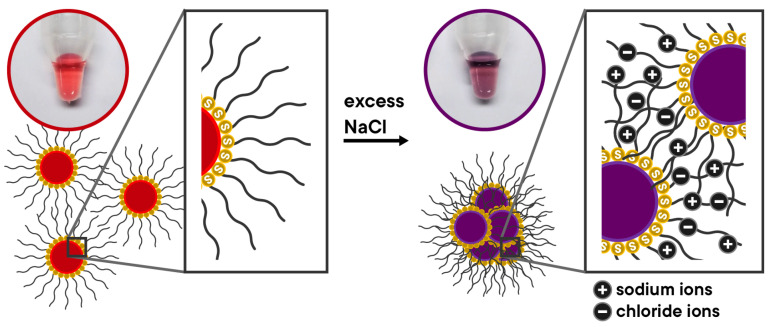
The mechanism of salt-induced aggregation of d-AuNP/DENV-2 probes.

The colorimetric detection method developed in this study is based on the salt-induced aggregation of d-AuNP/DENV-2 probes (Figure 3).

At high concentrations of NaCl, the d-AuNP/DENV-2 probes aggregate, resulting in a color change in the solution from red to violet. Initially, the d-AuNP/DENV-2 probes are repelled from each other due to the negatively charged phosphate backbone of the immobilized oligonucleotides on their surfaces, which keeps them dispersed. However, the addition of positively charged sodium ions (Na^+^) screens the negative charges (i.e., neutralized), reducing the repulsion between the probes. As a consequence, the colloidal solution becomes unstable, leading to aggregation. This process is characterized by a redshift in absorption from 520 nm to 620 nm and an increase in the degree of aggregation.

**Figure 4:**
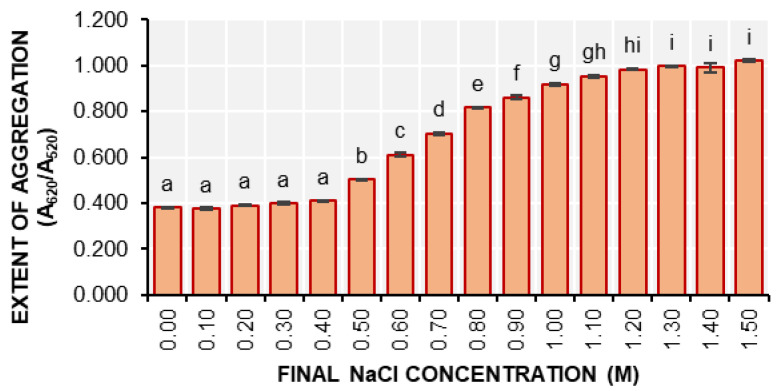
The extent of aggregation (A_620_/A_520_) of the 1.00 nmol d-AuNP/DENV-2 probes after the addition of the 0.10 to 1.50 M NaCl solutions (mean ± SD; *n* = 3). Samples with different letters on top of the bars are significantly different based on ANOVA and Tukey HSD test at α = 0.05.

The extent of aggregation of the 1.0 nmol d-AuNP/DENV-2 probes was assessed at various final NaCl concentrations (Figure 4). Based on the extent of aggregation at t = 15 min, it was found that the aggregation of the 1.0 nmol d-AuNP/DENV-2 probes could already occur even in NaCl concentrations as low as 0.50 M.

The aggregation response of this solution is significantly different (*p* < 0.05) from that of the probe solution without NaCl (0 M). Notably, the extent of aggregation for the 1.0 nmol d-AuNP/DENV-2 probe solution at a final concentration of 0.50 M was already significantly different from that at 0.00 M, even at the initial time point (t = 0 min). This suggests that aggregation occurs rapidly and may not be observed immediately. Consequently, the researcher decided to use 0.40 M NaCl as the final concentration for colorimetric detection instead of 0.50 M.

### 3.4. Colorimetric Detection of the Synthetic Oligonucleotide Targets

**Figure 5:**
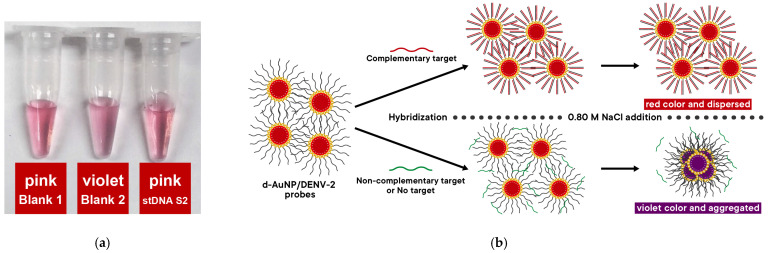
Colorimetric detection of the synthetic target DNA (stDNA S2) using the d-AuNP/DENV-2 probe: (**a**) colors of the solutions; (**b**) proposed mechanism of detection.

Synthetic target DNA (stDNA S2) designed for the d-AuNP/DENV-2 probes was used to test the nanobiosensor. Figure 5a shows the detection solutions 15 min after adding 0.80 M NaCl solution (resulting in a final NaCl concentration of 0.40 M). Visually, blank 2 appears violet, while stDNA S2 has the same pink color as blank 1 (which contains neither NaCl nor stDNA S2). This observation indicates that the probe successfully detected the presence of stDNA S2, as illustrated by the proposed mechanism in Figure 5b.

For the detection, the response was used as the parameter to indicate the presence of stDNA S2. It is the inverse of the extent of aggregation and is given by

**Figure 6:**
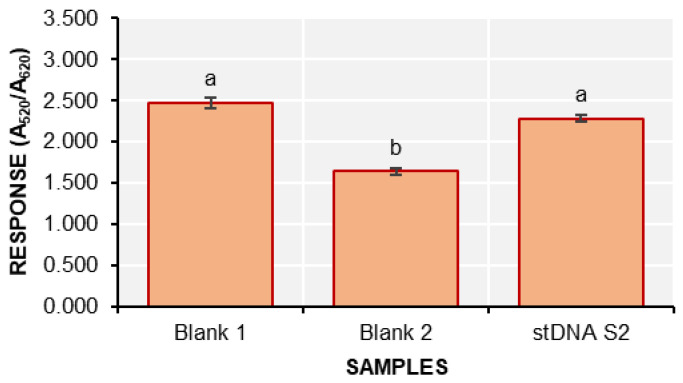
The response (A_520_/A_620_) of the samples during colorimetric detection using the d-AuNP/DENV-2 probe (mean ± SD; *n* = 3). Samples with different letters on top of the bars are significantly different based on ANOVA and Tukey HSD test at α = 0.05.

Response = A_520_/A_620_ (1)

where A_520_ is the absorbance at 520 nm and A_620_ is the absorbance at 620 nm. The responses of the detection solutions are shown in Figure 6.

The response of blank 2 is significantly different from both stDNA S2 and blank 1. This confirms that the nanobiosensor can indeed detect stDNA S2. However, it is important to note that the red and violet colors of the solutions are very faint, requiring a keen eye to clearly distinguish between the two.

### 3.5. Sensitivity of the Biosensor

**Figure 7:**
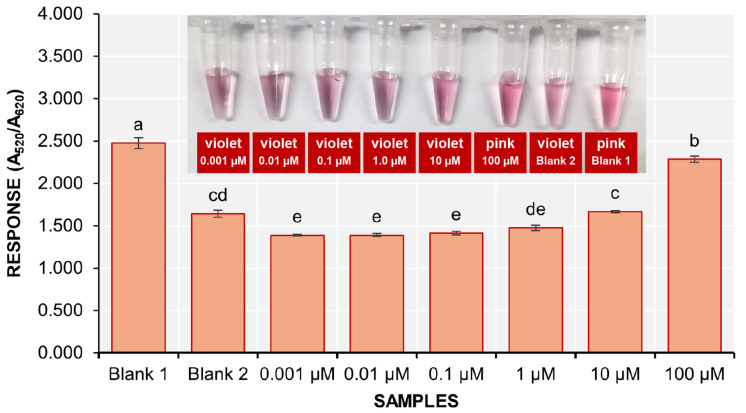
The response (A_520_/A_620_) of the samples during the determination of the sensitivity of the colorimetric detection method (mean ± SD; *n* = 3). Samples with different letters on top of the bars are significantly different based on ANOVA and Tukey HSD test at α = 0.05.

The sensitivity of the colorimetric detection assay was evaluated using serially diluted solutions of stDNA S2 at concentrations of 100, 10, 1.0, 0.10, 0.01, and 0.001 µM. The final concentrations of stDNA S2 in the detection solutions are 36.14, 3.62, 0.36, 0.036, 0.0036, and 0.00036 ng/µL, respectively. It was visually observed that the solutions containing 10, 1.0, 0.10, 0.01, and 0.001 µM had turned violet (Figure 7 inset).

The response of the 100 µM stDNA S2 solution was significantly different from that of blank 2, while the 10 and 1 µM stDNA S2 solutions did not show significant differences from blank 2. The remaining solutions displayed much lower responses compared to blank 2. Therefore, the nanobiosensor could only detect an initial concentration of 36.14 ng/µL of stDNA S2.

### 3.6. Specificity of the Biosensor

**Figure 8:**
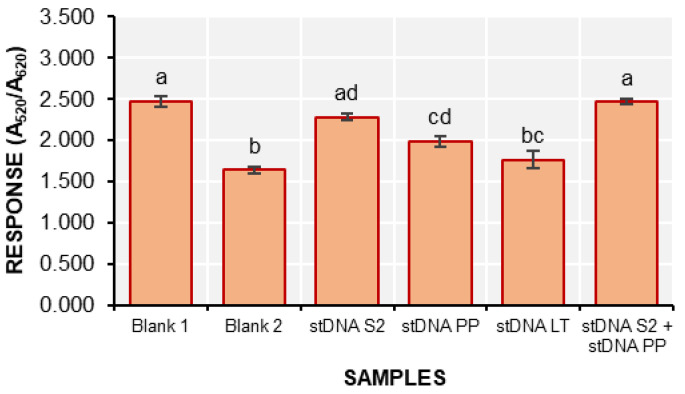
The response (A_520_/A_620_) of the samples during the determination of the specificity of the colorimetric detection method (mean ± SD; *n* = 3). Samples with different letters on top of the bars are significantly different based on ANOVA and Tukey HSD test at α = 0.05.

The specificity of the colorimetric detection assay was also assessed using two (2) other synthetic non-complementary target DNAs designed from *Phytophthora palmivora* (stDNA PP) and *Lasiodiplodia theobromae* (stDNA LT) (Figure 8).

The response of the stDNA S2 solution was not significantly different from that of the solutions containing stDNA PP and stDNA S2 + stDNA PP. In contrast, the response from the solution containing stDNA LT was significantly different from that of stDNA S2. This suggests that the d-AuNP/DENV-2 probe is not specific for stDNA S2 and shows cross-reactivity with stDNA PP.

## 4. Discussion

The d-AuNPs were prepared using the method developed by Anderson and colleagues [33]. This greener alternative to the traditional Turkevich (i.e., trisodium citrate method) and Brust–Schiffrin methods employs dextrin as the capping agent, with sodium carbonate serving as the reducing agent for the HAuCl_4_. They proposed that the alkaline synthesis of AuNPs with dextrin as a capping agent follows the mechanism outlined by Polte and colleagues in 2010 for the generation of citrate-capped AuNPs [40]. The mechanism involves a four-step growth mechanism: (1) reduction (sodium carbonate as reducing agent), (2) stabilization (mediated by oxidized carbonates), (3) exchange (slow growth), and (4) capping (fast growth).

In the first step, the Au^3+^ ions are reduced to Au^0^ by the carbonate ions acting as reducing agents (i.e., from Na_2_CO_3_). Upon dissolution in water, the HAuCl_4_ forms several complexes: [AuCl_4_]^−^, [AuCl_3_OH]^−^, [AuCl_2_(OH)_2_]^−^, [AuCl(OH)_3_]^−^, or [Au(OH)_4_]^−^. All of these complexes are ultimately reduced to Au^0^. In the second step, the oxidized carbonate stabilizes the initial AuNPs [33]. This initial nanoparticle formation is indicated by a visible color change in the solution from yellow to grayish. The third step represents a slow growth phase, characterized by exchanges between the dextrin and the carbonate ions occurring on the surface of the gold nanoparticles, which increases their stability. This phase is indicated by a purple tint in the solution. Finally, the reaction enters the fast growth phase, where dextrin is assumed to be the sole capping agent. This transition is visually observed as the color changes from purple to wine red. Furthermore, the researchers noted that smaller d-AuNPs were produced at higher concentrations of dextrin, illustrating a dependence of nanoparticle size on the concentration of the capping agent.

An important step in the preparation of detection probes for the colorimetric detection method is the formation of an Au-S covalent bond between the d-AuNPs and thiolated DNA probes. Thiolated DNA probes are not supplied as free thiols; instead, they exist in an oxidized form, where the sulfhydryl groups at the 5′-end or 3′-end are capped with a disulfide bond [41,42]. For this purpose, TCEP was used as the reducing agent to cleave this disulfide bond.

Both the d-AuNPs and DNA oligonucleotide probe carry net-negative charges. The d-AuNPs derive this from their dextrin capping agent and the negative charges on the gold surface, while the DNA oligonucleotides derive theirs from the negatively charged phosphate backbone. Typically, the d-AuNPs and DNA oligonucleotide probes repel each other. A salt such as NaCl could be used to overcome these long-ranged electrostatic repulsions [41]. The positively charged sodium ions (Na^+^) from NaCl screen these negative charges, allowing for the formation of the Au-S bond. However, it is crucial to note that the uncontrolled addition of salt may cause d-AuNP aggregation. By controlling the addition of salt, sufficient DNA can be gradually immobilized on the surface of the d-AuNPs, further stabilizing the nanoparticles through steric stabilization and stronger electrostatic repulsion [41].

**Figure 9:**
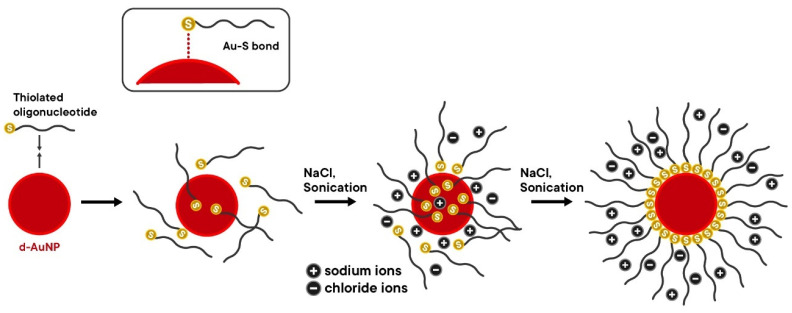
The mechanism of the salt-aging method during the immobilization of the oligonucleotides on the d-AuNPs’ surface.

The salt-aging method developed by Hill and Mirkin (2006) was modified by Hurst and colleagues (2006) to add more steps to the process [32,35]. This method addresses the problem of AuNP aggregation that can occur when 1 M salt is added all at once to the conjugation reaction solution. The presence of PAB helps to prevent drastic changes in the pH of the reaction mixture. On the other hand, Liu and Liu (2017) discussed that the addition of surfactants such as SDS increases DNA loading efficiency by 39%. This improvement is achieved through the formation of an interdigitated bilayer structure that allows the thiol terminal end of the DNA oligonucleotide probe to slowly enter this bilayer and adsorb onto the d-AuNP surface [41]. Figure 9 shows the mechanism of the salt-aging method.

Once NaCl is added, some of the DNA oligonucleotide probes attach to the surface of the d-AuNPs, which increases the net-negative charge on the d-AuNP surface and further hinders the attachment of incoming probes. The stability of the d-AuNPs also improves due to the initial attachment of probe molecules. As the NaCl concentration increases with successive additions, more DNA probe molecules become adsorbed on the d-AuNP surface until a new electrostatic repulsion equilibrium is established. The additional DNA probes enhance the stability of the d-AuNPs at higher NaCl concentrations. It is important to mention that some dextrin capping agents are displaced during this process, as the formation of Au-S bonds is more favorable than the adsorption of dextrin onto the d-AuNPs. As the salt-aging process continues, the thiol groups gradually displace the DNA bases already adsorbed on the d-AuNP surface, allowing them to assume an upright position relative to the nanoparticle’s surface. Brief sonication after every addition of NaCl was also proven to improve the DNA loading efficiency by decreasing the non-specific interactions between the DNA bases and the d-AuNP surface [32,41].

The proposed mechanism of detection of the nanobiosensor in the presence of complementary and non-complementary targets is shown in Figure 5. In the absence of NaCl, the d-AuNP/DENV-2 probes remain dispersed in the solution. In the presence of a complementary target DNA (i.e., stDNA S2), it hybridizes with the DNA oligonucleotide probes to form duplexes based on the Watson–Crick base pairing model. As such, the duplex assumes a more linear conformation and further increases the effective negative charge on the surface of the d-AuNPs. Consequently, the d-AuNP/DENV-2 probe/stDNA S2 complex becomes more stable compared to the unhybridized d-AuNP/DENV-2 probes. Thus, when NaCl is added, the d-AuNP/DENV-2 probe/stDNA S2 complex remains dispersed, and the red color of the solution does not change. Conversely, when a non-complementary target DNA is present, hybridization does not occur, meaning no duplexes are formed on the d-AuNP surface. Therefore, upon the addition of NaCl, the d-AuNP/DENV-2 probe becomes unstable, leading to aggregation. In this case, the color of the solution changes from red to violet. In summary, a positive result for this nanobiosensor is indicated by the retention of the red color of the d-AuNP/DENV-2 probe, while a shift to violet signals a negative result.

Since the goal of the nanobiosensor is to detect only a single serotype (DENV-2), care was taken in the design of the oligonucleotide sequence to ensure specificity for DENV-2. Therefore, utilizing the Watson–Crick model of base pairing for complementary oligonucleotide sequences is critical for achieving high specificity in DENV-2 detection. However, it was observed that one of the non-complementary targets (i.e., stDNA PP) showed cross-reactivity with the designed probe. This suggests that some sequences in the designed probe may be complementary to the sequences found in stDNA PP. To enhance the specificity of the biosensor, we recommend using the actual sequence of genomic RNA from viral isolates for designing the DNA probes, rather than solely relying on sequences from online databases.

Additionally, issues encountered during the immobilization of the DNA probes on the surface of the d-AuNPs could be addressed by employing 11-mercaptoundecanoic acid (MUDA)-functionalized d-AuNPs and 5′-aminated DNA probes. Furthermore, alternative methods to the salt-aging process could be explored, such as surfactant-assisted, low-pH-assisted, and depletion stabilization techniques.

## 5. Conclusions

In this study, we optimized the reaction conditions for the DNA-based colorimetric detection of the synthetic target oligonucleotide for DENV-2. The d-AuNPs were successfully prepared using the method of Anderson and colleagues (2011). UV-Vis spectrophotometric analysis revealed that the wine-red d-AuNP solution had a wavelength of maximum absorption (λ_max_) of approximately 520 nm. We successfully immobilized the 1.0 nmol thiolated DENV-2 probes on the surface of the d-AuNPs using the salt-aging method, resulting in the formation of d-AuNP/DENV-2 probes. Additionally, we conducted salt-induced aggregation experiments, which showed that a minimum final concentration of 0.40 M NaCl is required to initiate aggregation of the d-AuNP/DENV-2 probes. The colorimetric detection was successfully performed, demonstrating that the biosensor could detect synthetic complementary target stDNA S2 at concentrations up to 36.14 ng/μL, with some cross-reactivity observed with one non-complementary target. To enhance the specificity of the developed nanobiosensor, we recommend designing the probe based on the actual sequence of the genomic RNA from viral isolates, rather than solely using sequences found in online databases. Furthermore, the specificity of the designed nanobiosensor could be evaluated against other DENV serotypes. We also suggest that colorimetric detection be performed on viral genomes isolated from mosquito samples to assess the performance of this nanobiosensor. Finally, we also recommend the assessment of the linearity and reproducibility of the responses being obtained from the developed nanobiosensor. Our study provides a basis for the development of nanobiosensors targeting other DENV serotypes.

## Figures and Tables

**Table 1:** Summary of different biosensors developed to detect DENV.

Transducer	Bioreceptor	Material(s) Used	Serotype(s) and Sample	Limit of Detection	Reference
impedimetric	lectin	Fe_3_O_4_ nanoparticles	DENV-1, -2, and -3 in sera	3.09 nM	[20]
potentiometric	antibody	alumina-modified electrodes	DENV-2 in mosquito	1 pfu/mL	[21]
amperometric	antibody	carbon nanotube	DENV-1 in human sera	12 ng/mL	[22]
potentiometric	oligonucleotide	electrically active magnetic (EAM) nanoparticles	DENV-2 in mosquito	10 ng/μL	[19]
SPR sandwich assay	antibody	silver nanoparticles (AgNPs)	DENV-1	150 ng/mL	[23]
LSPR; aggregation-based	oligonucleotide	DNAzymes coupled to AuNPs	DENV-3	5 × 10^2^ PFU/mL	[15]
SPR sandwich assay	oligonucleotide	lateral flow biosensor with AuNPs	DENV-1	1.2 × 10^4^ PFU/mL	[3]

**Table 2:** The composition of the reaction solutions for the colorimetric detection of stDNA S2 using the d-AuNP/DENV-2 probe.

Sample	Volume of Component (μL)				
	1× sPBS pH 7.4	sdH_2_O Type 1	1.0 nmol d-AuNP/ DENV-2 Probe	100 µM Synthetic Targets	0.80 M NaCl Solution
Blank 1	10	80	10	0	0
Blank 2	10	30	10	0	50
stDNA S2	10	25	10	5	50
stDNA PP *	10	25	10	5	50
stDNA LT *	10	25	10	5	50
stDNA PP and stDNA PP *	10	20	10	5	50

* Non-complementary synthetic target DNA: (a) stDNA PP (*Phytophthora palmivora*) and (b) stDNA LT (*Lasiodiplodia theobromae*).

## Data Availability

The data presented in this study are available upon request from the corresponding author.

## Supplementary Materials

The following supporting information can be downloaded at https://www.mdpi.com/article/10.3390/bios15020071/s1: Table S1: List of *Aedes aegypti* DENV-2 DNA sequences retrieved from NCBI for multiple sequence alignment.
