# Supplementary material for: DNA-Based Nanobiosensor for the Colorimetric Detection of Dengue Virus Serotype 2 Synthetic Target Oligonucleotide
Source: Biosensors (Basel). 2025 Jan 24;15(2):71. doi: 10.3390/bios15020071 (PMC11853087; doi:10.3390/bios15020071)
Supplement: Supplementary file 1 [file biosensors-15-00071-s001.zip › biosensors-3322208-supplementary.pdf]

SUPPORTING INFORMATION

DNA-Based Nanobiosensor for the Colorimetric Detection of Dengue Virus Serotype 2 Synthetic Target Oligonucleotide

Michael Sandino C. Flores [1,*], Evangelyn C. Alocilja [2,3], Divina M. Amalin [4,5], Mae Joanne B. Aguila [1], Marynold V. Purificacion [1,6], Florinia E. Merca [1], Ma. Carmina C. Manuel [7], Mark Pierre S. Dimamay [8], Ma. Anita M. Bautista [9] and Lilia M. Fernando [1,10,*]

[1] Institute of Chemistry, College of Arts and Sciences, University of the Philippines Los Baños, College, Los Baños 4031, Philippines; mbaguila@up.edu.ph (M.J.B.A.); mvpurificacion@up.edu.ph (M.V.P.); femerca@up.edu.ph (F.E.M.)
[2] Nano-Biosensors Lab, Biosystems and Agricultural Engineering, Michigan State University, East Lansing, MI 48824, USA; alocilja@msu.edu
[3] Global Alliance for Rapid Diagnostics, Michigan State University, East Lansing, MI 48824, USA
[4] Department of Biology, College of Science, De La Salle University Manila, Taft Avenue, Malate, Manila 1004, Philippines; divina.amalin@dlsu.edu.ph
[5] Institute of Biological Control, De La Salle University-Laguna Campus, Biñan City 4024, Philippines
[6] Institute of Plant Breeding, College of Agriculture and Food Science, University of the Philippines Los Baños, College, Los Baños 4031, Philippines
[7] Genetics and Molecular Biology Division, Institute of Biological Sciences, College of Arts and Sciences, University of the Philippines Los Baños, College, Los Baños 4031, Philippines; mcmanuel1@up.edu.ph
[8] Research and Biotechnology Division, St. Luke's Medical Center, Quezon City 1112, Philippines; mpsdimamay@stlukes.com.ph
[9] Functional Genomics Laboratory, National Institute of Molecular Biology and Biotechnology, University of the Philippines Diliman, Quezon City 1101, Philippines; mmbautista20@up.edu.ph
[10] Institute of Crop Science, College of Agriculture and Food Science, University of the Philippines Los Baños, College, Los Baños 4031, Philippines
* Correspondence: mcflores4@up.edu.ph (M.S.C.F.); lmfernando@up.edu.ph (L.M.F.)

Table S1. List of *Aedes aegypti* DENV-2 DNA sequences retrieved from NCBI for multiple sequence alignment.

COUNTRY OF ORIGIN	ACCESSION NUMBER	LENGTH (BASES)
India	FJ538906	2541
	FJ538919	2541
Thailand	HQ379625	118
	JN692493	1056
	JQ846016	342
	JN692493	1056
	JQ846016	342
Pakistan	KF186663	340
India	KF850531	384
Thailand	KM003971	306
	KM003972	306
	KM003973	306
	KM003974	306
	KM003975	306
	KM003976	306
	KM003977	306
	KM003978	306

	KM003979	306
	KM003980	306
	KM003981	306
	KM003982	306
India	KM507029	479
	KR706555	289
	KR706556	289
	KR706557	289
	KT327919	233
	KT345604	286
	KT345605	363
	KT345606	317
	KT345607	260
	KT345608	250
	KT345610	528
	KT345612	519
	KT345613	347
Thailand	KY234157	1068
	KY234171	1485
	KY234186	300
	LC030023	345
	LC030024	345
	LC030025	345
	LC030026	345
	LC030027	345
	LC030028	345
	LC030029	345
	LC030030	345
	LC030031	345
	LC030032	345
	LC030033	345
	LC030034	345
	LC030035	345
	LC030036	345
	LC030037	345
	LC030038	345
	LC030039	345
	LC030040	345
	LC030041	345
	LC030042	345
	LC030043	345

	LC030044	345
	LC030045	345
	LC030046	345
	LC030047	345
	LC030048	345
	LC030049	345
	LC030050	345
Philippines	MK268743	676
	MK268747	279
	MK268748	684
	MK268749	278
	MK268750	684
	MK268752	685
Sri Lanka	MT256401	1485
